# Intra- and interobserver variability of left ventricular diastolic inflow parameters measured by three chamber view 2D cine three-directionally encoded (3Ch. 2D-cine-3 dir.) Phase contrast MR velocity vector map

**DOI:** 10.1186/1532-429X-15-S1-P127

**Published:** 2013-01-30

**Authors:** Munemura Suzuki, Masashi Sakuma, Norihiko Kotooka, Takuya Ueda, Hiroshi Suito, Hiroyuki Irie, Koichi Node

**Affiliations:** 1 Department of Radiology, Takagi Hospital, Okawa, Japan; 2 Department of Cardiology, Saga University Hospital, Saga, Japan; 3 Department of Radiology, Saga University Hospital, Saga, Japan; 4 Department of Radiology/Cardiovascular Center, St. Luke's International Hospital, Tokyo, Japan; 5 Graduate school of environmental and life science, Okayama University, Okayama, Japan

## Background

One-directionally-encoded cine 2D Phase-Contrast MRI (PC MRI) of basal short axial image is generally used for the evaluation of diastolic function, but has potential limitations due to the longitudinal cardiac motion and inflow direction. Time-resolved 3D PC MRI is not affected by the flow direction and left ventricular motion, however, requires long acquisition time. In theory, three chamber view 2D cine three directionally-encoded (3ch. 2D-cine-3dir.) PC MR velocity vector map could overcome such limitations. The aim of this study is to assess intra- and interobserver variability of diastolic parameters measured by 2D-cine-3dir. PC MR velocity vector map.

## Methods

**Figure 1:**
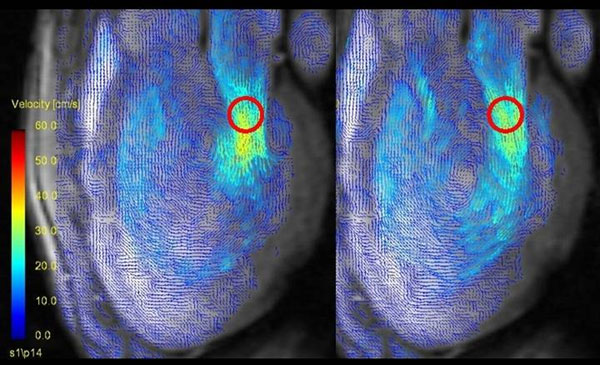
3Ch. 2D-3dir. MR velocity vector maps at the early (left) and late (right) diastolic inflow. Red circles indicate ROIs

Retrospectively gated 3ch. 2D-cine-3dir. PC MRI was performed on 32 patients (F:M = 13:19, Mean age: 59.4 ± 14 years) for various indication. Velocity encoding were set from 150 to 300 cm/sec to avoid aliasing. Two independent observer generated 3ch. velocity vector map from three phase images and one magnitude image on commercially available MR flow data analysis software. First, anatomical structures and left ventricular inflow pattern (two peaks or single peak) were identified on the map (Figure 1). Then, a round ROI was placed between the mitral tips at each time flame and the peak E and A velocity were measured. One reader repeated the same procedure twice on the other day. Intra- and interobserver variability were calculated by means of intraclass correlation coefficient (ICC (1,1) and ICC (2,1)) for the peak E and A velocity, Spearman's rank correlation coefficient for E/A ratio, and Cohen's kappa coefficient for inflow pattern.

## Results

All the PC MRI examination were successfully obtained in one breath hold and analyzed within 3 minutes. Both intra- and interobserver variability for the peak E, A velocity, E/A ratio and flow pattern showed almost perfect agreement or very strong correlation, except for the peak E velocity (ICC (2,1) = 0.751) and flow pattern (Cohen's kappa = 0.788) (Table 1).

**Table 1:** 

	E velocity ICC(95%CI)	A velocity ICC(95%CI)	E/A ratio (Spearman's rho)	Inflow pattern Cohen's kappa
Intraobserver variability	0.929 (0.86-0.964)	0.976 (0.943-0.990)	0.839	1

Interobserver variability	0.751 (0.534-0.876)	0.970 (0.921-0.989)	0.844	0.788

## Conclusions

3ch. 2D-cine-3dir. PC MR velocity vector map is a highly reliable and reproducible method and could be used as a part of routine examination.

## Funding

This research is conducted under the support of JST's CREST funding program.

